# Grids in Topographic Maps Reduce Distortions in the Recall of Learned Object Locations

**DOI:** 10.1371/journal.pone.0098148

**Published:** 2014-05-28

**Authors:** Dennis Edler, Anne-Kathrin Bestgen, Lars Kuchinke, Frank Dickmann

**Affiliations:** 1 Department of Geography, Ruhr-University Bochum, Bochum, Germany; 2 Department of Psychology, Ruhr-University Bochum, Bochum, Germany; University of Leicester, United Kingdom

## Abstract

To date, it has been shown that cognitive map representations based on cartographic visualisations are systematically distorted. The grid is a traditional element of map graphics that has rarely been considered in research on perception-based spatial distortions. Grids do not only support the map reader in finding coordinates or locations of objects, they also provide a systematic structure for clustering visual map information (“spatial chunks”). The aim of this study was to examine whether different cartographic kinds of grids reduce spatial distortions and improve recall memory for object locations. Recall performance was measured as both the percentage of correctly recalled objects (hit rate) and the mean distance errors of correctly recalled objects (spatial accuracy). Different kinds of grids (continuous lines, dashed lines, crosses) were applied to topographic maps. These maps were also varied in their type of characteristic areas (LANDSCAPE) and different information layer compositions (DENSITY) to examine the effects of map complexity. The study involving 144 participants shows that all experimental cartographic factors (GRID, LANDSCAPE, DENSITY) improve recall performance and spatial accuracy of learned object locations. Overlaying a topographic map with a grid significantly reduces the mean distance errors of correctly recalled map objects. The paper includes a discussion of a square grid's usefulness concerning object location memory, independent of whether the grid is clearly visible (continuous or dashed lines) or only indicated by crosses.

## Introduction

The cognition of geographic space is a topic attracting researchers from various disciplines. The vast majority of research into spatial cognition has been directed towards the acquisition, encoding, storing, recalling and decoding of spatial information [1], [2]. These psychological fundamentals, however, have hardly been exploited to upgrade the geographical perspective on space – especially when it comes to the question of how to design useful and effective graphic replica of the topographic reality [3], [4].

Object-location memory is fundamental for investigations of spatial cognition. When people learn locations of objects, they abstract the spatial structure of a layout in terms of a spatial reference system [5], [6] to form a mental representation or cognitive map of the environment [7]. Such cognitive maps can be derived from the environment (direct experience) or from working with graphic media representing the environment (indirect experience), i.e. maps or map-like visualisations [8]. Thus, cognitive maps involve information about spatial objects, relations and distances between objects as well as the absence of objects and other information gaps [9]–[11]. There is some evidence that cognitive maps based on indirect experiences are prone to systematic distortions [12]–[14]. These consistent distortions are premised on hierarchical (top-down) processing of spatial information [15]–[19].

In addition to spatial distortions caused by hierarchical top-down coding, the majority of systematic distortions in spatial cognition based on indirect experiences are thought to have perceptual origins. These distortions reflect the principles of perceptual grouping [20] and hierarchical encoding of spatial information [7], [21]–[24]. It is known from vision research that such perceptual organization has early beginnings [14], [25]. It likely starts with the first fixation on a visual scene [26]–[28]. Therefore, it is assumed that perception- and memory-based processes influence spatial memory in a conjoint fashion [29]. The map reader splits the spatial layout into a set of perceptual object units (spatial “chunks”). This perception-based chunking is guided by structuring map elements that ‘regionalize’ the map and supports learning of objects and their spatial relations [21], [23], [30]. Here, learning refers to a superordinate framework consisting of specific graphic features, such as grid lines or map-inherent features [17]. These graphic features create a functional structure, such as road, railway and river systems or coordinate grids. They represent spatial information aggregates of the map contents. These spatial information aggregates, on the other hand, are structured hierarchically and are as such essential parts of cognitive maps [17], [18], [31]. Accordingly, recent evidence from vision research reveals that structuring map elements, such as grids, assist both the perception and recognition of object locations [32]–[34]. For example, Stainer and colleagues [35] were able to show that a grid structure on a visual scene changes eye-movement fixation patterns from having a central bias to quadrant-based central biases. Again, this effect can be shown to start with the first fixation on a scene.

In cartography, empirical studies on map perception or design are of increasing importance (see especially [36]–[40]). Cartographers often emphasise that empirical research has so far hardly addressed systematic examinations of cartographic elements in terms of the formation of cognitive maps [3], . Given that topographic maps as used in cartography differ from abstract maps (i.e. blank maps that often comprise only black lines and simple object symbols), at least in terms of visual complexity, the question arises whether the displayed information can be used to reduce systematic distortions of spatial knowledge about the displayed environment. For example, square grids overlaid on topographic maps are commonly used in a traditional cartographic or geodetic context, i.e. to define coordinates or to find locations of map objects [43]–[45].

The function of grids as structuring map elements in topographic maps has hardly been explored so far. A standard approach to investigate spatial memory is the recall paradigm when people are asked to recall previously learned object locations from maps or map-like visualizations [5], [14], [18], [24], [46]–[48]. For example, it was recently shown that compared to blank maps, continuous grid lines improve the recall of object locations learned from these maps (as indicated by reduced distances from the original locations, [49]). Similarly, already the presentation of a topographic base layer (i.e. a sketch of physical height information) reduces the mean distance error. Adding grid lines to a physical base layer does not lead to further improvement in recall performance [49]. Both map features add visual complexity to a map. By adding visual details, these map elements support the regionalization of a visual scene and provide reference objects that can be used to encode categorical spatial relations [50]. Thus, it has been argued that complex maps, such as urban maps, support the formation of more accurate cognitive maps due to their higher number of reference nodes [51]. In vision research, linearly increasing effects of the amount of distinct visual details (i.e. ‘clutter’ [52]) on visual search times for targets in a scene are reported [53], but a relation to object-location memory has not yet been examined.

Other authors suggested that spatial distortions are greater in cognitive maps of complex, especially urban, topographies [54]–[56]. Similarly, cognitive load theory [57], [58] assumes that our cognitive system seeks to avoid redundant information. For example, from a cartographic perspective, the user is at the heart of the map design process [59]–[63]. Hikers, bikers and travellers need different information when making themselves familiar with an area's topography [64]. Thus, going along with cognitive load theory, information layers not directly supporting the users should be avoided. It is suggested that avoiding redundant information in the construction of maps reduces the mental effort [64], [65] and may therefore improve the encoding of spatial relations. As a consequence, different base map topographies varying in their visual complexity and different layers of map information should be considered when investigating the effects of grids on spatial memory.

**Figure 1:**
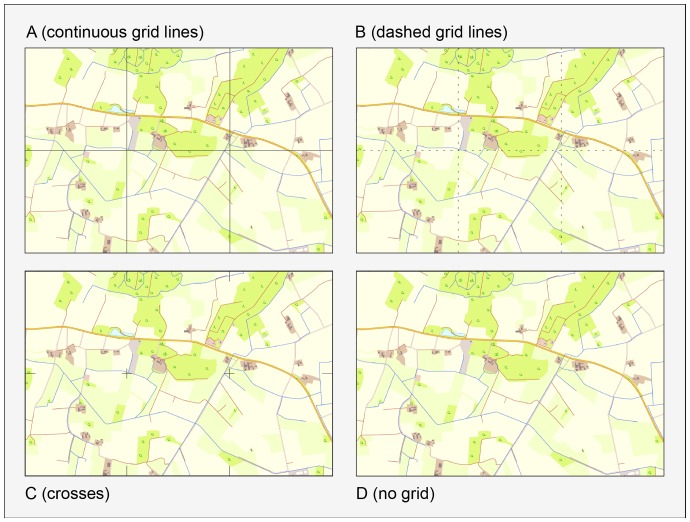
The four kinds of grids. Each map was featured with four different kinds of grids (upper left: continuous lines, upper right: dashed lines, lower left: crosses, lower right: no grid). The example used in this illustration is the map of Sendenhorst, Germany – derived from ATKIS-Basis-DLM (cf. Figure 2).

One aim of the present study was to examine the effects of different grids on object-location memory in a recall paradigm as measured by the percentage of correctly recalled object locations and their average deviation from the original object location (or mean distance error). The factor GRID refers to different visualizations of grids (e.g., continuous lines, dashed lines, crosses, cf. Figure 1) as used in cartography [43], adding different levels of complexity to the map. In addition to GRID, the map-inherent factors LANDSCAPE (5 levels) and DENSITY (3 levels, nested within LANDSCAPE, see Methods) were used in this study to investigate topographic base maps with different levels of visual complexity. Summarizing the above literature (e.g., [4], [49]) it was expected that both the grids and the more complex topographic visualizations improve object-location memory.

## Methods

The study was conducted in accordance with the Declaration of Helsinki and approved by the local ethics committee of the Faculty of Psychology, Ruhr-University Bochum (Germany). All participants gave their written informed consent prior to being included in the study.

### Participants

One hundred forty-four participants (62 male, 82 female) aged between 19 and 35 [M = 24.3; SD  = 3.0] participated in the study for pay. All participants were unaware of the study purpose and reported having normal vision or corrected-to-normal vision. All participants were students at the Ruhr-University Bochum. They were unfamiliar with the topography represented in the study materials.

### Materials

Sixty-four different digital maps were created as study materials. The scale of each map was 1/10,000. This map scale is ideal for users dealing with, for instance, route planning, travel management, city maps and tourism – i.e. user groups dealing with wayfinding and navigation issues [66]. The sixty-four maps were subdivided into four sets of sixteen maps differing in the type of GRID: continuous grid lines (A), dashed grid lines (B), crosses (C), no grid (D) (see Figure 1). A, C and D are common design patterns used by cartographers when they make maps [43]. A continuous grid is defined as a possible map feature in official German 1/10,000 scale topographic maps [67]. Apart from type D, the grid layers contained or indicated continuous compositions of equidistant and parallel lines forming identical square cells. Each grid cell covered an area of 1 km^2^. The sizes of the cells were based on the principle of spacing with horizontal and vertical grid lines each 1,000 meters on a 1/10,000 map [43], [45]. The grid layer was spatially adjusted so that it created six square cells, three in the horizontal plane multiplied by two in the vertical direction. Compared to type A, type C has the aesthetic advantage of causing less overlap with other map objects [43], [68]. Type C indicates the chunking of the map surface into squares, whereas type A involves continuous squares. Type B is closely related to a so-called “rouletted grid” [45] in which the lines are composed of closely-spaced dot-like units. At present, this is a quite unusual alternative in mapmaking [45]. However, it is a solution that decreases the overlap compared to type A and emphasises the square structure more than type C. Matching the proposal of the ATKIS-catalogue for map graphics [67], the colour of the grids was black (R: 0, G: 0, B: 0). The line width was 0.5 pt/0.18 mm and thus matched the cartographic guideline of using either dark brown or black lines of 0.1–0.2 mm gauge [45].

**Figure 2:**
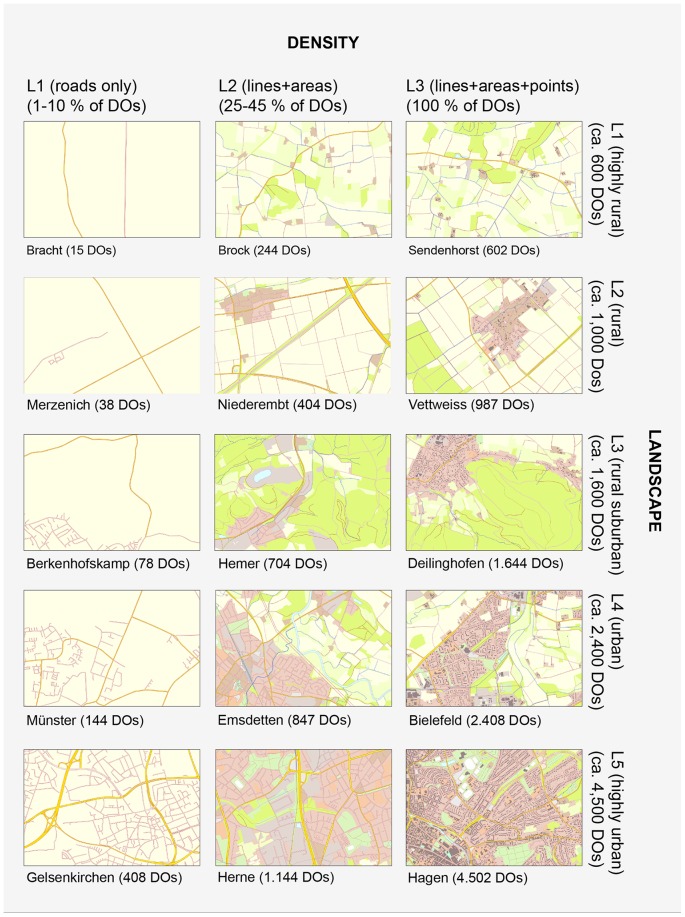
The fifteen maps derived from ATKIS-Basis-DLM. Each map had a unique combination of DENSITY and LANDSCAPE. The three levels of DENSITY refer to the categories of active object layers. Each level of DENSITY represents a different percentage range of the total number of distinct objects (DOs) −1–10%, 25–45% or 100%. The five levels of LANDSCAPE (highly rural, rural, rural-suburban, urban, highly urban) refer to categories of characteristic topographies as used in cartography. The caption of each map refers to the represented area of Germany's federal state of North Rhine-Westphalia.

In addition, two factors of map-inherent complexity were selected: landscape category (LANDSCAPE) and information layer density (DENSITY). Sixteen maps (15 main trials and one practice trial) of each set represented the topography of sixteen different places in North-Rhine Westphalia (NRW), Germany. NRW covers an area of about 35,000 km^2^ and includes rural and mountainous areas as well as areas of high urban density [69]. This topographic diversity was considered in the qualitative map-inherent factor LANDSCAPE, representing five categories of characteristic types of topography: highly rural, rural, rural-suburban, urban, highly urban (Figure 2). Map complexity can also be measured by the total number of distinct objects (DOs) displayed [70], [71]. Therefore, the five categories are also defined quantitatively by a sufficient number of DOs when considering all object information currently displayed in an ATKIS-based map, excl. verbal elements. The approximate numbers of DOs for the five categories were 600 (highly rural), 1,000 (rural), 1,600 (rural-suburban), 2,400 (urban) and 4,500 (highly urban). The number of DOs was determined using the multiresolution segmentation algorithm [72] and further image classification methods implemented in the geographic object-based image analysis (GEOBIA) software Trimble eCognition Developer 8.7.2. The segmentation and classification considered spectral pixel values (coloured) as well as the shape and compactness of image information to determine homogeneous image objects. The identified numbers of distinct objects characterising LANDSCAPE was double-checked with ArcMap 10.1, the main component of Esri's ArcGIS suite of geospatial processing programs. Alternatively, map complexity can be determined by lossless JPEG compressed file lengths [52], [74]. On average, the following jpg file sizes were obtained: 1,036 kb (highly rural), 1,564 kb (rural), 1,784 kb (rural-suburban), 2,552 kb (urban) and 4,261 kb (highly urban).

The DENSITY factor refers to the layer composition of the displayed content with three levels nested within each level of LANDSCAPE. The first level comprises roads only. By limiting the original composition to the road layers, approximately 10 percent (or less) of the original amount of DOs is displayed. The second level extends the idea of a road map and comprises roads as well as different categories of its surrounding areas (different land use classes, such as parks, forests and industrial areas). This level covers approximately 25–45 percent of the original amount of DOs. The third level comprises all point, linear and area elements contained in the graphics of the contemporary and ATKIS-based digital topographic map of North-Rhine Westphalia (DTK10-V-NRW), including the outline of each building [73] (i.e. 100% of the DOs, cf. Figure 2). To examine DENSITY, each of the maps assigned to one of the five categories of LANDSCAPE were further modified according to one specific level of DENSITY. In this way, all fifteen maps were featured with a unique combination of LANDSCAPE and DENSITY. Please note that LANDSCAPE and DENSITY are not independent and because the DENSITY factor is nested within the levels of LANDSCAPE in the present design, it qualifies the computation and interpretation of interaction terms between these factors.

The to-be-learned object locations were six circular symbols representing the locations of places of interest (POIs) on each map. Care was taken not to position POIs at the boundary of grid borders in order to avoid confounding factors (e.g., based on the route effect [22]). The same POI positions were used in all four grid conditions. The distribution of the POIs and the sequence of presenting the maps were random. All symbols were identical in size (d = 1 cm) and colour (R: 225, G: 0, B: 200). Any verbal elements, such as written place names and other object/attribute labels, were removed from the maps. All maps used in this study were derived from official geodata sets acquired and maintained by German public authorities (ATKIS-Basis-DLM). The study maps were mainly created using *ArcMap 10.1*. The grids were added using the vector graphics editor *Adobe Illustrator CS 5*. The final maps were then embedded into a script tool based on *Adobe Flash CS 5*. This script was used to run the trials and to acquire all test data needed. The maps were displayed on a TFT-LCD 24” screen that was calibrated in order to represent map colours designed in Germany [73].

### Procedure

The study consisted of a three-factorial 3*5*4 mixed design comprising the within-subjects factors DENSITY (3) and LANDSCAPE (5), while GRID (4) was the between-subjects factor. The participants were randomly assigned to one of four GRID groups. Each participant took part in 15 study-test trials in random order. In each trial, they were shown one of the fifteen study maps for 60 s and were asked to memorise the locations of the six POIs. The study phase was immediately followed by a filler task (60 s of multiplication exercises). After that, the map was shown again for 60 s without POIs, and the participants were asked to recall the six POI locations. To solve the recall task, the participants were instructed to use the mouse cursor and were asked to place the recalled POIs on the map. The participants were allowed to shift the locations of each point until they confirmed the position. Prior to the first 15-minute test trial, the participants were given a practice trial to make themselves familiar with the software, the tasks, and the general test procedure. The participants were encouraged to complete their tasks as accurate as possible.

### Analyses

The recall performance was assessed by measuring the Euclidean distance between the x and y coordinates of the recalled places of interests (POI) and the corresponding coordinates of its original location. The distance was measured in pixels (px). In accordance with previous research [4], [13], [49], the location of a recalled POI was considered correct if it differed no more than 28.4 px (0–1 cm) from the original location. A hierarchical 3*5*4 ANOVA including the within-subjects factors DENSITY and LANDSCAPE as well as the between-subjects factor GRID was computed for the hit rate (percentage of correctly recalled POIs) and spatial accuracy (mean distance errors of correctly recalled POIs). In the hierarchical ANOVA model, DENSITY was defined as nested within LANDSCAPE. The significance threshold was set at p = .05. The violation of the assumption of sphericity was tested by Mauchly's test, and Greenhouse-Geisser-correction was applied if appropriate. Significant main effects were further examined by Bonferroni-corrected pairwise comparisons; only significant results were reported. Significant two-way interactions were resolved by linear and/or quadratic contrast after splitting the data by one of the factors to reveal linear and/or quadratic patterns in the data contributing to the particular interaction. Responses were considered for the ANOVAs only when at least two correctly remembered POIs increased the participant's average score. Due to this constraint, the data of 47 participants could not have been considered for further analyses. This high drop-out rate is probably linked to the low 1 cm criterion in the present analysis. Overall, in 1.5% of all trials, 0 POIs were correctly recalled (1 POI: 4.3%, 2 POIs: 8.6%, 3 POIs: 13.2%, 4 POIs: 17.1%, 5 POIs: 21.9%, 6 POIs: 33.5%). Of note is that the distribution differed between the grid conditions, with the ‘no grid’ condition showing the highest amount of trials not reaching the 1 cm criterion (no grid: 62 trials, crosses: 12 trials, dashed lines: 17 trials, continuous lines: 33 trials). A chi-square test confirmed that the number of trials not reaching the 1 cm criterion significantly differed across the levels of GRID (chi-square (3)  = 52.09, p<.001).

Although it was not the main aim of the present study, sex differences as a between-subject factor were included in an additional analysis. Previous research on spatial abilities revealed that males tend to outperform females, for instance, in virtual maze tasks and in learning from navigation in a space [75]–[79]. The superior performance by males does not refer to all tasks, but this is typical when people learn spatial configurations in the environment (direct experience). When they learn from maps (indirect experience), no differences between males and females are observed [80]. Several studies indicated that females show equal (e.g. [81]) or better recall performance than males [82]–[86]; see also [46], [87]. Of note is that sex differences in recalling object locations from official topographic maps have not been tested so far.

## Results

### Hit rates

**Figure 3:**
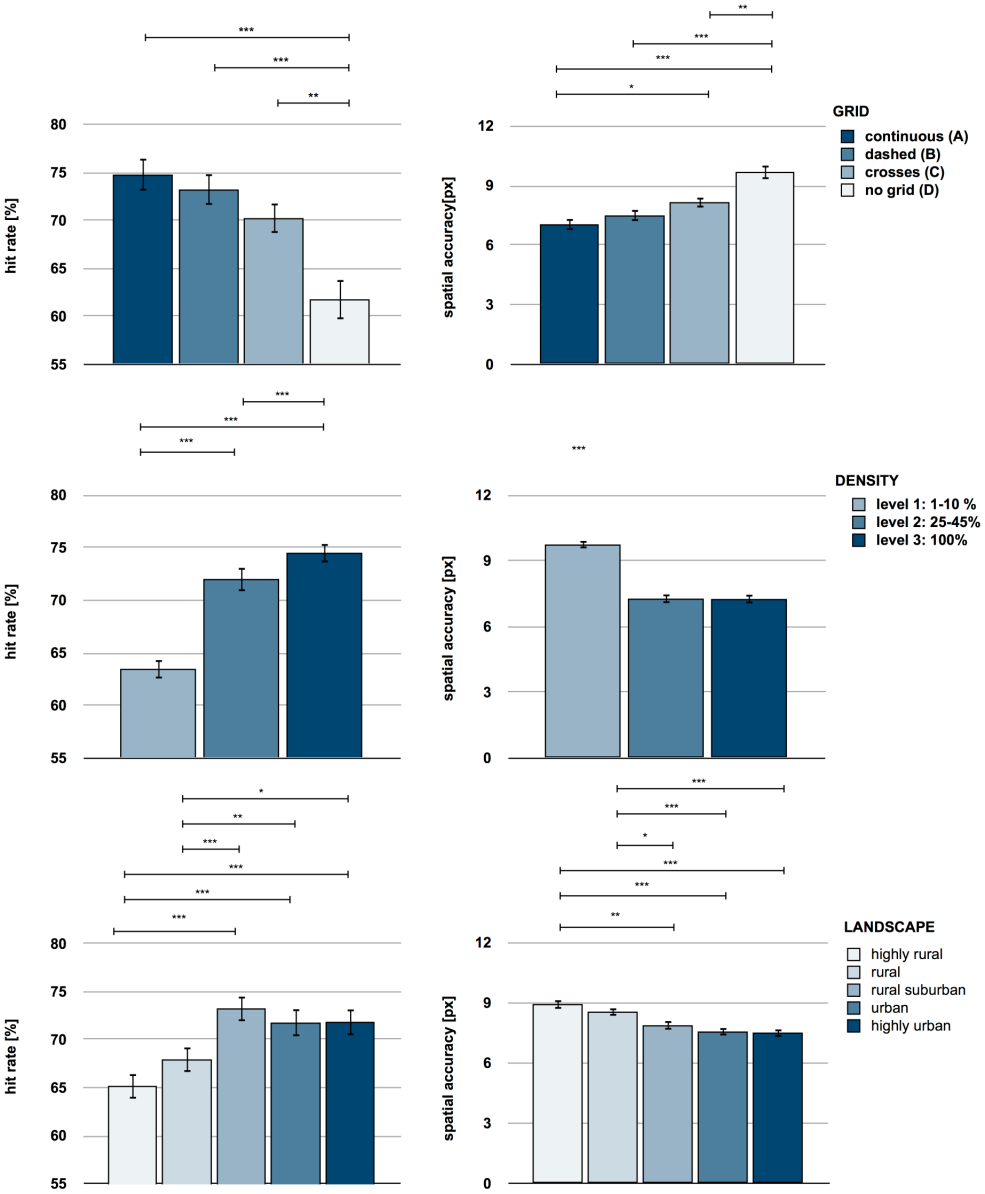
Main effects of the experimental factors (GRID, LANDSCAPE, DENSITY) on hit rate and spatial accuracy. Hit rate refers to the percentage of correctly recalled places of interest (POIs); spatial accuracy represents the mean distance errors of correctly recalled POIs (in px). The recall of a POI was considered as correct if the recalled location was within a linear distance of 0–28.4 px (0–1 cm) from the location of the original object. Error bars represent the standard error of the mean. *  =  p<.05; **  =  p<0.01; ***  =  p<.001 (Bonferroni-corrected).

The ANOVA on the percentage of correctly recalled POIs revealed significant main effects of GRID (*F*(3,93)  = 8.981, *p*<.001, *η^2^*  = .26), LANDSCAPE (*F*(4,372)  = 22.034, *p*<.001, *η^2^*  = .19) and DENSITY (*F*(8,744)  = 10.393, *p*<.001, *η^2^*  = .101) (Figure 3). In addition, the significant interaction GRID*LANDSCAPE (*F*(12,372)  = 2.131, *p* = .015, *η^2^*  = .64) was observed, whereas GRID*DENSITY (F(24,744)  = 1.151, p = .280, η^2^  = .036) was not significant.

**Figure 4:**
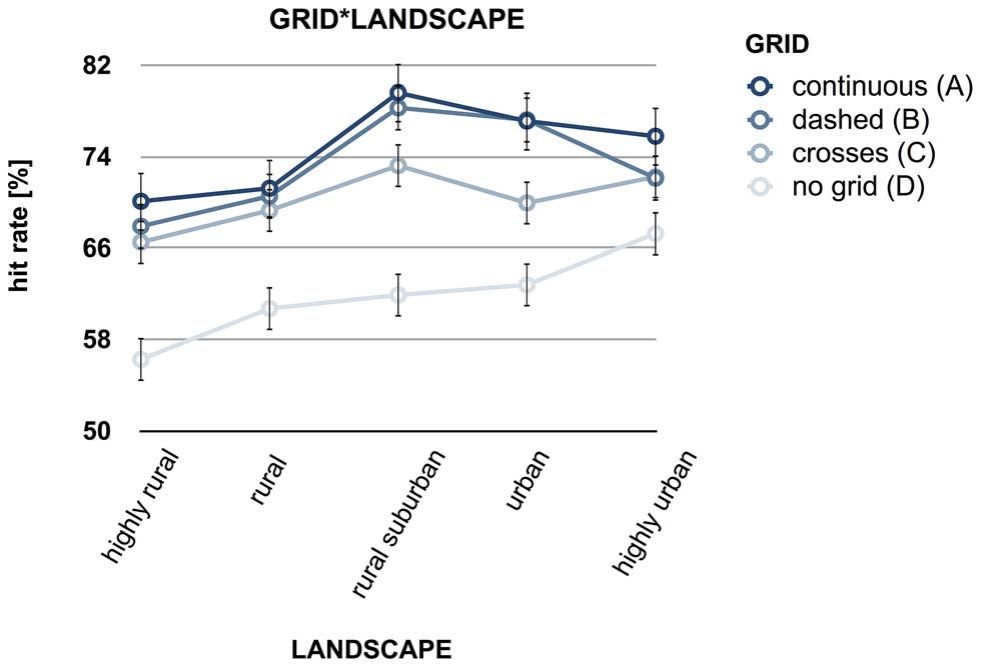
Interaction of GRID and LANDSCAPE on hit rate. Hit rate refers to the percentage of correctly recalled places of interest (POIs). Significant linear trends are identified for all four kinds of grids. Continuous (A) and dashed grids (B) also reveal a significant quadratic trend (at p<.05).

Post hoc comparisons for GRID revealed that the hit rate for maps without grids (m = 61.78%) was significantly lower compared to maps with grids (all p's<.009, continuous: m = 74.78%, dashed: m = 73.78%, crosses: m = 70.24%). No significant differences were observed between the continuous, dashed and crossed GRID levels. The post-hoc examination of LANDSCAPE revealed that hit rates for highly rural (m = 65.18%) and rural (m = 67.19%) maps were significantly lower than the hit rates for all other levels of LANDSCAPE (all p's<.011, see Figure 3). No significant differences between highly rural and rural (p = .064), and between rural-suburban (m = 73.26%), urban (m = 71.78%) and highly urban (m = 71.86%) were revealed. In terms of DENSITY, hit rates at level 1 were significantly lower than at level 2 and level 3 (all p's<.001), while the hit rates at level 2 were significantly lower than level 3 (p<.001). In terms of GRID*LANDSCAPE interaction, each level of GRID showed linearly increasing contrast (all p's<.017). In addition, the quadratic contrast reached significance (p's<.05) in terms of continuous (F(1,24)  = 26.699) and dashed grid lines (F(1,26)  = 22.113, see Figure 4).

### Spatial accuracy

The mixed ANOVA on the mean distance errors of correctly recalled POIs revealed significant main effects of GRID (*F*(3,93)  = 11.849, *p*<.001, *η^2^*  = .28), LANDSCAPE (F(4,372)  = 13.906, *p*<.001, *η^2^*  = .13) and DENSITY (F(8,744)  = 13.187, p<.001, *η^2^*  = .124) (Figure 3), with no interaction term reaching significance (p's >.27). The main effect of GRID was based on a significantly higher mean distance error in maps without grids (*m* = 9.68 px) than maps with grids (all p's<.007). Furthermore, the mean distance error for crosses (m = 8.15 px) was significantly higher than for continuous grid lines (m = 7.05 px, p = .037, Figure 3). Pairwise comparison for the main effect of LANDSCAPE revealed that the mean distance error was higher in highly rural (m = 8.94 px) and rural maps (m = 8.56 px) compared to all other categories (all p's<.046, rural-suburban: m = 7.88 px, urban: m = 7.57 px, highly urban: m = 7.52 px). No significant differences between highly rural and rural maps, or between rural-suburban, urban and highly urban maps were observed. The main effect of DENSITY was based on a higher mean distance error at level 1 compared to level 2 (p<.001) and level 3 (p<.001), whereas the last two did not differ.

### Female vs. male participants

In a separate analysis, the factor SEX was added to the analyses of hit rates and spatial accuracy. No significant main effect of SEX on hit rates (*F*(1,95)  = .028, *p*  = .868, *η^2^* <.001) or spatial accuracy (*F*(1,95)  = .592, *p* = .444, *η^2^*  = .006) was observed. The average hit rate was 82.43% (SD  = 1.77) for male participants and 82.81% (SD  = 1.42) for female participants. The mean distance error was 8.10 px (SD  = .27) for male participants and 7.83 px (SD  = .22) for female participants. The factor SEX did not interact with any of the main effects or interactions comprising GRID, DENSITY and LANDSCAPE in terms of hit rates (p's >.192) and spatial accuracy (p's >.624), whereas the result pattern of the analyses described above was replicated.

## Discussion

Encoding and recall of object locations in complex topographic maps (scale: 1/10,000) covering different land use was affected by the map-inherent factors of LANDSCAPE and DENSITY, and the addition of GRIDs. Any type of GRID (continuous lines, dashed lines and crosses) overlaid on these maps significantly increased both hit rates and spatial accuracy compared to ‘no grid’ condition, whereas differences between these GRID levels were scarce. Grids help improve spatial memory, which confirms the main hypothesis of this study. It is pointed out that the uniform structure of grid cells provides the map users with spatial chunks of information and apparently supports the encoding and recall of object locations. This result replicates previous findings in vision research on the effect of grid structure on spatial recall [32]–[34] and transfers these findings to the idea of memory effects in complex topographic maps. It is well known that square cells support the visual inspection of scenes [35]. Grid cells as a focus for the map reader supplements the established geodetic and geographic functions of map grids (i.e. to define coordinates or to find locations of map objects [43]–[45]), and is a supporting tool for object-location memory. Based on the present results, it seems likely that during the encoding of an object location, people focus on the information contained in a square cell to improve encoding and subsequent memory retrieval.

Strong effects of GRID (continuous lines, dashed lines or crosses) are visible compared to the ‘no grid’ condition, but barely differ between each other. Continuous lines, dashed lines and crosses all seem to support the hierarchical structuring of the map into information aggregates [34], [35]. They influence memory positively and reduce distortions in cognitive representations [17], [18], [31]. The spatial accuracy measure shows an advantage of continuous grid lines over crosses, which is of particular note. The more solid grid lines are, the more the deviation of a recalled location decreases. It seems likely that the more the effect of grids on object-location memory increases, the more obvious they appear in the map. Although visible grid lines add further visual detail and thus complexity to a map, they still increase recall performance (Figure 3), which contradicts the assumptions of cognitive load theory [57], [58]. It is rather suggested that grids form reference objects to encode spatial relations [50], [51].

An enhancement of the recall performance with higher complexity is also shown in terms of hit rate and spatial accuracy of the nested factors DENSITY and LANDSCAPE. The recall performance increases with a higher availability of distinct objects (DOs) – most visible in a linear parametric increase of the hit rates for DENSITY (Figure 3). The effects of DENSITY for both hit rate and spatial accuracy indicate a strong performance decline in level 1. If a map contains main roads only, the mental representation is characterised by greater distortions than at the other two levels. In terms of hit rate, the integration of buildings (level 3), as currently recommended by public authorities [73], improves object-location memory, most likely due to anchor effects of reference nodes in urban topographies [51]. Of note is that this result, at first glance, seems to contradict cognitive load theory and assumptions that complex, heavily urbanised maps would entail greater need for spatial orientation [60]; see also [54], [56] and more recognition errors in case of increasing visual complexity [53], [74]. An explanation for these diverging findings is provided by the ‘levels of processing’ [88], where it has been proven that a higher demand during memory encoding leads to better memory performance. In the present study, map encoding based on higher DENSITY might have had a similar effect on spatial memory.

The effect on spatial accuracy was less pronounced, but the two higher levels of DENSITY improved the participants' spatial accuracy, compared to low DENSITY maps with main roads only. In contrast to suggestions for user-centred map design [63]–[65], the removal of (redundant) map information, i.e. applying lower DENSITY, does not necessarily improve information processing in maps. Although higher visual complexity is expected to increase visual search times [53], it also decreases distortions of learned object locations. The removal of detailed representation of houses does not influence spatial accuracy. Still, both dependent measures indicate that not all map information (like urban areas and DOs) is redundant.

The results of the factor LANDSCAPE replicate and extend this assumption: more urban areas comprising a greater amount of DOs increase hit rate and spatial accuracy. Overall, the results of LANDSCAPE, with nested DENSITY, also question the parametric linearity of these relationships. Figure 3 reveals that the effects of the more urban levels of LANDSCAPE on hit rate is rather curvilinear, as it is also indicated by the significant quadratic contrasts contributing to the GRID*LANDSCAPE interaction of continuous and dashed grid lines (cf. Figure 4). The curvilinearity of these effects might indicate an optimum (or ceiling effect) of increasing LANDSCAPE levels (please also note a similar curvilinear effect for DENSITY in Figure 3), when an addition of DOs to a complex map does not further increase the amount of correctly recalled objects (also [49]). It seems likely that an addition of DOs might even (slightly) decrease recall performance (see above [64], [65]). This curvilinear trend matches findings of aesthetics research into visual complexity of images [89], [90] and contradicts studies on visual search in which linear parametric effects are reported (on search time and eye movement scan paths depending on visual complexity) [52], [53], [91]. A linear relationship cannot be simply transferred to spatial memory. Figure 4 indicates a linear trend in maps without grids where the hit rate increases from rural to urban topographies: the more complex the LANDSCAPE level of the displayed topography, the better the hit rate. Adding grid lines leads to the curvilinear relationship, though still at a higher level of performance compared to the ‘no grid’ condition. Although grids decrease spatial distortions (thus leading to better formation of a cognitive map) throughout the levels of LANDSCAPE, it becomes obvious that urban topographic maps gain a smaller benefit from grids than more rural topographies. This leaves open the possibility that the effects of topography and grid lines are not additive. It can be speculated that the higher amount of DOs reduces the impact of spatial chunking by square grid cells.

This is the first study documenting these complex interactions. Future examinations manipulating visual features in maps might help to advance our understanding of these effects, and in particular of whether an optimum of available map information can be quantified and replicated with other complex map material. Still, it should also be noted that this is the first study to reveal that map-inherent topographic features of LANDSCAPE and DENSITY, and cartographic features like different kinds of GRIDS contribute to a reduction of spatial memory distortions. All three factors guide memory performance and affect spatial memory in an interactive manner. Thus, the cartographer, who is responsible for map graphics, is able to influence distortion tendencies in the user's spatial memory. Assuming the spatial quality of a cognitive map is important for map use (e.g. object-location memory when navigating), the representation of a given LANDSCAPE can be optimised by providing a GRID (continuous, dashed or crossed) and a high level of DENSITY.

### Sex differences

Contrary to some results of previous research into sex differences in object-location memory where female participants showed superior performance (e.g. [82]–[86]), the analysis performed within this study does not reveal any significant difference between female and male recall performance (see also [81]). It is suggested that women and men use different strategies in spatial orientation. For example, women refer to local landmarks whereas men have a tendency to rely on environmental geometries and metric distances [79], [80], [92]. As this is beyond the scope of the present study, we can only assume that the different strategies by the different sexes result in equal performance quality when encoding and recalling objects in topographic maps.

### Limitations and future directions

Three limitations of the present study should be mentioned. First, the design did not allow a separate analysis and discussion of encoding and retrieval effects. We believe that with the help of eye-tracking methods, future studies will be able to examine the encoding phase more deeply and thus be able to separate the effects grids and map complexity have during encoding and recall. Second, the topographic base layer was varied using a nested factor: the three levels of DENSITY co-occured within each level of LANDSCAPE. This hierarchical approach limits the interpretation of DENSITY*LANDSCAPE interactions. However, the approach copes with user-centred design issues in cartography. The factor DENSITY offers the option to select information layer compositions according to the individual purpose of map use. The activation of useful and de-activation of redundant information layers are issues often discussed in map design (e.g. [59]–[63]). Based on the available maps, a fully orthogonal design seems difficult to construct. Third, each combination of LANDSCAPE and DENSITY was represented by only one specific map. Follow-up studies should include more maps per cell in the design to be able to average across maps and thus increase generalizability of the results by diminishing map-specific effects. Overall, the strong statistical effects indicate a robustness of the observed effects.

## Conclusion

Object-location memory in topographic maps is influenced by different factors that add complexity to a map. A layer of square grids increases recall performance. Object-location memory is poorer in maps involving no grids, whereas different kinds of grids help reduce distortions. These results refer to topographic base maps featured with varying topographic bases. A topographic base showing a rural area provides less support for the formation of a more detailed and accurate cognitive representation of the area than a topography including more urban features, whereas a map that shows only the main roads of an area causes weaker recall performance than maps containing additional point and area features. The data are in line with the assumption that a higher amount of visual objects adds frames of reference to maps that guide spatial memory. An increase in redundancy cannot explain these data, and theoretical proposals are not supported by the present data. To further support theory formation in cartography, the next step would be the examination of the individual map parameters and features, such as the ideal sizes of grid cells. Future studies geared towards the definition of the graphic grid parameters should also consider aesthetic aspects of mapmaking. From a traditional cartographic perspective, it is most appreciated to maintain a low design profile of artificial map features, especially when they overlap other map objects [93]. Therefore, the minimum size of effective kinds of grids should be determined.

## References

[1] Downs RM, Stea D (1973) Image and environment: cognitive mapping and spatial behaviour. Chicago: Aldine Publishing Company. 439 p.

[2] Wessel G, Unruh E, Sauda E (2013) Heads up: using cognitive mapping to develop a baseline description for urban visualization. In: Proceedings of the Sixth International Conference on advances in Computer-Human Interactions (ACHI), 24 February –1 March. Nice (France), 6 p.

[3] Fabrikant SI, Lobben A (2009) Cognitive issues in geographic information visualisation. Cartographica 44: 139–143.

[4] Dickmann F, Edler D, Bestgen A, Kuchinke L (2013) Spatial distortions in cognitive maps – a chance and challenge to enrich the principles of map design. Kartographische Nachrichten 63: 174–181.

[5] McNamara TP, Valiquette CM (2004) Remembering where things are. In: Allen GL, editor. Human Spatial Memory. Remembering Where. Mahwah: Lawrence Erlbaum Associates, Publishers. 3–24.

[6] Tversky B (2003) Structures of mental spaces. How people think about space. Environ Behav 35: 66–80.

[7] Tversky B (1993) Cognitive maps, cognitive collages, and spatial mental models. In: Frank AU, Campari I, editors. Spatial information theory: a theoretical basis for GIS. Berlin: Springer. 14–24.

[8] Dickmann F (2012) City maps versus map-based navigation systems – an empirical approach to building mental representations. Cartogr J 49: 62–69.

[9] Barkowsky T (2002) Mental representations and processing of geographic knowledge. A computational approach. Berlin: Springer. 188 p.

[10] Mark DM, Freksa C, Hirtle SC, Lloyd R, Tversky B (1999) Cognitive models of geographical space. Int J Geogr Inf Sci 13: 747–774.

[11] O'Keefe J, Nadel L (1978) The hippocampus as a cognitive map. Oxford: OUP. 504 p.

[12] Tversky B (1992) Distortions in cognitive maps. Geoforum 23: 131–138.

[13] Okabayashi H, Glynn SM (1984) Spatial cognition: systematic distortions in cognitive maps. J Gen Psychol 111: 271–279. 6512518 10.1080/00221309.1984.9921116

[14] Tversky B (1981) Distortions in memory for maps. Cogn Psychol 13: 407–433.

[15] Baylis GC, Driver J (1993) Visual attention and objects: evidence for hierarchical coding of location. J Exp Psychol Hum Percept Perform 19: 451–470. 8331310 10.1037//0096-1523.19.3.451

[16] McNamara TP, Hardy JK, Hirtle SC (1989) Subjective hierarchies in spatial memory. J Exp Psychol Learn Mem Cogn 15: 211–227. 2522511 10.1037//0278-7393.15.2.211

[17] Eastman RJ (1985) Graphic organization and memory structures for map learning. Cartographica 22: 1–20.

[18] Hirtle S, Jonides J (1985) Evidence of hierarchies in cognitive maps. Mem Cognit 13: 208–217. 10.3758/bf03197683 4046821

[19] Stevens A, Coupe P (1978) Distortions in judged spatial relations. Cogn Psychol 10: 422–437. 699514 10.1016/0010-0285(78)90006-3

[20] Coren S, Girgus JS (1980) Principles of perceptual organization and spatial distortion: the gestalt illusions. J Exp Psychol Hum Percept Perform 6: 404–412. 6447756 10.1037//0096-1523.6.3.404

[21] Hurts K (2005) Common Region and Spatial Performance Using Map-like Displays. In: Proceedings of the Human Factors and Ergonomics Society Annual Meeting: 1593–1597.

[22] Klippel A, Knuf L, Hommel B, Freksa C (2004) Perceptually induced distortions in cognitive maps. Spatial Cognition IV: Reasoning, Action, Interaction: 204–213.

[23] Hommel B, Gehrke J, Knuf L (2000) Hierarchical coding in the perception and memory of spatial layouts. Psychol Res 64: 1–10. 11109863 10.1007/s004260000032

[24] McNamara TP, Ratcliff R, McKoon G (1984) The mental representation of knowledge acquired from maps. J Exp Psychol Learn Mem Cogn 10: 723–732. 6239008 10.1037//0278-7393.10.4.723

[25] Montello DR (1998) Kartenverstehen: Die Sicht der Kognitionspsychologie. Zeitschrift für Semiotik 20: 91–103.

[26] Oliva A, Torralba A (2006) Building the gist of a scene: the role of global image features in recognition. Prog Brain Res 155: 23–36. 17027377 10.1016/S0079-6123(06)55002-2

[27] Henderson JM (2003) Human gaze control during real-world scene perception. Trends Cogn Sci 7: 498–504. 14585447 10.1016/j.tics.2003.09.006

[28] Biederman I, Mezzanotte RJ, Rabinowitz JC (1982) Scene perception: detecting and judging objects undergoing relational violations. Cogn Psychol 14: 143–177. 7083801 10.1016/0010-0285(82)90007-x

[29] Eastman JR (1985) Cognitive models and cartographic design research. Cartogr J 22: 95–101.

[30] Clements-Stephens AM, McKell-Jeffers GO, Maddux J-M, Shelton AL (2011) Strategies for spatial organization in adults and Children. Vis cogn 19: 886–909.

[31] McNamara TP (1986) Mental representations of spatial relations. Cogn Psychol 18: 87–121. 3948491 10.1016/0010-0285(86)90016-2

[32] Leifert S (2011) The influence of grids on spatial and content memory. In: Proceedings of the 2011 annual conference extended abstracts on Human factors in computing systems (Vancouver, BC, Canada: ACM): 941–946.

[33] Wolfe JM, Võ ML-H, Evans KK, Greene MR (2011) Visual search in scenes involves selective and nonselective pathways. Trends Cogn Sci 15: 77–84. 21227734 10.1016/j.tics.2010.12.001 PMC3035167

[34] Martin R, Houssemand C, Schiltz C, Burnod Y, Alexandre F (2008) Is there continuity between categorical and coordinate spatial relation coding? Evidence from a grid/no-grid working memory paradigm. Neuropsychologia 46: 576–594. 18037455 10.1016/j.neuropsychologia.2007.10.010

[35] Stainer MJ, Scott-Brown KC, Tatler BW (2013) Behavioral biases when viewing multiplexed scenes: scene structure and frames of reference for inspection. Front Psychol 4: 624. 24069008 10.3389/fpsyg.2013.00624 PMC3781347

[36] Stachoň Z, Šašinka Č, Štěrba Z, Zbořil J, Březinová Š, et al (2013) Influence of graphic design of cartographic symbols on perception structure. Kartographische Nachrichten 63: 216–220.

[37] Weninger B (2013) The effects of colour on the interpretation of official noise maps. In: Proceedings of 26th International Cartographic Conference ICC 2013 (Dresden, Germany). ICC website. Available: http://www.icc2013.org/_contxt/_medien/_upload/_proceeding/285_proceeding.pdf. Accessed 2014 May 5.

[38] Skiles MD, Howarth JT (2012) From signs to minds: spatial information design and mental maps. Cartogr J 49: 312–325.

[39] Bunch RL, Lloyd RE (2000) The search for boundaries on maps: color processing and map pattern effects. Cartogr Geogr Inf Sci 27: 15–29.

[40] Bollmann J (1981) Aspekte kartographischer Zeichenwahrnehmung. Eine empirische Untersuchung. Bonn: Kirschbaum. 264 p.

[41] Griffin AL, Fabrikant SI (2012) More maps, more users, more devices means more cartographic challenges. Cartogr J 49: 298–301.

[42] Kettunen P, Irvankoski K, Krause CM, Sarjakoski T, Sarjakoski LT (2012) Geospatial images in the acquisition of spatial knowledge for wayfinding. Journal of Spatial Information Science 5: 75–106.

[43] Hake G, Grünreich D, Meng L (2002) Kartographie. 8th ed. Berlin: de Gruyter. 604 p.

[44] Robinson AH, Morrison JL, Muehrcke PC, Kimerling AJ, Guptill SC (1995) Elements of cartography. 6^th^ ed. New York: Wiley. 688 p.

[45] Maling DH (1992) Coordinate systems and map projections. 2^nd^ ed. Oxford: Pergamon. 476 p.

[46] Lloyd RE, Bunch RL (2008) Explaining map-reading performance efficiency: gender, memory, and geographic information. Cartogr Geogr Inf Sci 35: 171–202.

[47] Uttal DH (2000) Seeing the big picture: map use and the development of spatial cognition. Dev Sci 3: 247–286.

[48] Montello DR, Sullivan CN, Pick HL (1994) Recall memory for topographic and natural terrain: effects of experience and task performance. Cartographica 31: 18–36.

[49] Bestgen A, Edler D, Dickmann F, Kuchinke L (2013) Grid or no grid: distance distortion in recognizing spatial information from complex cartographic maps. In: Proceedings of CogSci 2013–35^th^ Annual Meeting of the Cognitive Science Society, (Berlin, Germany). MindModeling@Home website. Available: http://mindmodeling.org/cogsci2013/papers/0062/paper0062.pdf. Accessed 2014 May 5.

[50] Kosslyn SM (1987) Seeing and imagining in the cerebral hemispheres: a computation-al approach. Psychol Rev 94: 148–175. 3575583

[51] Golledge RG (1991) Cognition of physical and built environments. In: Gärling T, Evans GW, editors. Environment, cognition and action – an integrated approach. New York: OUP. 35–62.

[52] Rosenholtz R, Li Y, Nakano L (2007) Measuring visual clutter. J Vis 7(2): 1–17. 10.1167/7.2.17 18217832

[53] Neider MB, Zelinsky GJ (2011) Cutting through the clutter: searching for targets in evolving complex scenes. J Vis 14(7): 1–16. 10.1167/11.14.7 22159628

[54] Lloyd R (1989) Cognitive maps: encoding and decoding information. Ann Assoc Am Geogr 77: 191–207.

[55] Matthews MH (1985) Young children's representation of the environment: a comparison of techniques. J Environ Psychol 5: 261–278.

[56] Evans GW, Skorpanich MA, Gärling T, Bryant K, Bresolin B (1984) The effects of pathway configuration, landmarks, and stress on environmental cognition. J Environ Psychol 4: 323–335.

[57] DeLeeuw KE, Mayer RE (2008) A comparison of three measures of cognitive load: Evidence for separable measures of intrinsic, extraneous, and germane load. J Educ Psychol 100: 223–234.

[58] Chandler P, Sweller J (1996) Cognitive load while learning to use a computer program. Appl Cogn Psychol 10: 151–170.

[59] Meng L (2013) Cartography and maps beyond disciplines. Kartographische Nachrichten 63: 115–122.

[60] Westerbeek H, Maes A (2013) Route-external and route-internal landmarks in route descriptions: effects of route length and map design. Appl Cogn Psychol 27: 297–305.

[61] Speake J, Axon S (2012) “I never use maps anymore”: engaging with sat nav technologies. Cartogr J 49: 326–336.

[62] Crampton JW (2009) Cartography: maps 2.0. :Prog Hum Geogr 33: 91–100.

[63] Gartner G, Bennett DA, Morita T (2007) Towards ubiquitous cartography. Cartogr Geogr Inf Sci 34: 247–257.

[64] Tversky B, Agrawala M, Heiser J, Lee P, Hanrahan P, et al.. (2006) Cognitive design principles: from cognitive models to computer models. In: Magnani L, editor. Model-Based Reasoning in Science and Engineering. London: College Publications. 1–20.

[65] Davies C, Uttal DH (2007) Map use and the development of spatial cognition. In: Plumert JM, Spencer JP, editors. The emerging spatial mind. Oxford: OUP. 219–247.

[66] Bezirksregierung Köln (2013) Digitale Topographische Karte 1: 10.000 (DTK10). Bezirksregierung Köln website. Available: http://www.bezreg-koeln.nrw.de/brk_internet/organisation/abteilung07/produkte/topographisch/dtk10/index.html. Accessed 2014 Mar 28.

[67] AdV (2010) Amtliches Topographisch Kartographisches Informationssystem. ATKIS. Technisches Regelwerk für den Datenaustausch von Rasterdaten der Topographischen Karten. Version 1.8. AdV website. Available: http://www.adv-online.de/icc/extdeu/med/658/65870a72-255b-df11-a3b2-1718a438ad1b,11111111-1111-1111-1111-111111111111. Accessed 2014 March 28.

[68] Goodenough W, MacLeod M, McCaw GT, Hinks AR, Winterbotham HSL (1933) The use of the new grid on Ordnance Survey maps: discussion. Geogr J 82: 47–54.

[69] Wolf N, Edler D, Jürgens C (2011) A spatiotemporal analysis of Germany's largest urban agglomeration. In: Proceedings of the 2011 RSPSoc Annual Conference (Bournemouth, UK): 8 p.

[70] Harrie L, Stigmar H (2009) An evaluation of measures for quantifying map information. ISPRS J Photogramm Remote Sens 65: 266–274.

[71] Schnur S, Bektas K, Salahi M, Çöltekin A (2010) A comparison between of measured and perceived visual complexity for dynamic web maps. In: Proceedings of the Sixth International Conference on Geographic Information Science. (Zurich, Switzerland). giscience2010 website. Available: http://www.giscience2010.org/pdfs/paper_181.pdf. Accessed 2014 Mar 28.

[72] Baatz M, Schaepe A (2000) Multiresolution segmentation – an optimization approach for high quality multi-scale image segmentation. In: Strobl J, Blaschke T, Griesebner G, editors. Angewandte Geographische Informationsverarbeitung XII. Karlsruhe: Wichmann. 12–23.

[73] Landesvermessungsamt NRW (2005) Auszug aus dem Zeichenmuster der DTK10-V-NRW. Cologne: 6 p.

[74] Donderi DC, McFadden S (2005) Compressed file length predicts search time and errors on visual displays. Displays 26: 71–78.

[75] Hegarty M, Montello DR, Richardson AE, Ishikawa T, Lovelace KL (2006) Spatial abilities at different scales: individual differences in aptitude-test performance and spatial-layout learning. Intelligence 34: 151–176.

[76] Waller D (2000) Individual differences in spatial learning from computer-simulated environments. J Exp Psychol Appl 6: 307–321. 11218340 10.1037//1076-898x.6.4.307

[77] Lawton CA, Morrin KA (1999) Gender differences in pointing accuracy in computer-simulated 3D mazes. Sex Roles 40: 73–92.

[78] Montello DR, Lovelace KL, Golledge RG, Self CM (1999) Sex-related differences and similarities in geographic and environmental spatial abilities. Ann Assoc Am Geogr 89: 515–534.

[79] Sandstrom NJ, Kaufman J, Huettel SA (1998) Males and females use different distal cues in a virtual environment navigation task. Brain Res Cogn Brain Res 6: 351–360. 9593991 10.1016/s0926-6410(98)00002-0

[80] Wolbers T, Hegarty M (2010) What determines our navigational abilities? Trends Cogn Sci 14: 138–146. 20138795 10.1016/j.tics.2010.01.001

[81] Lloyd RE, Bunch RL (2005) Individual differences in map reading spatial abilities using perceptual and memory Processes. Cartogr Geogr Inf Sci 32: 33–46.

[82] Voyer D, Postma A, Brake B, Imperato-McGinley J (2007) Gender differences in object location memory: a metaanalysis. Psychon Bull Rev 14: 23–38. 17546728 10.3758/bf03194024

[83] Rahman Q, Wilson GD, Abrahams S (2003) Sexual orientation related differences in spatial memory. J Int Neuropsychol Soc 9: 376–383. 12666762 10.1017/S1355617703930037

[84] Dabbs JM, Chang E, Strong RA, Milun R (1998) Spatial ability, navigation strategy, and geographic knowledge among men and women. Evol Hum Behav 19: 89–98.

[85] Tottenham L, Saucier D, Elias L, Gutwin C (2003) Female advantage for spatial location memory in both static and dynamic environments. Brain Cogn 53: 381–383. 14607186 10.1016/s0278-2626(03)00149-0

[86] Galea LAM, Kimura D (1993) Sex differences in route-learning. Pers Individ Dif 14: 53–65.

[87] Lloyd RE, Bunch RL (2010) Learning geographic information from a map and text: learning environment and individual differences. Cartographica. 45: 169–184.

[88] Craik FIM, Lockhart RS (1972) Levels of processing: a framework for memory research. J Verbal Learning Verbal Behav 11: 671–684.

[89] Hekkert P, van Wieringen PCW (1990) Complexity and prototypicality as determinants of the appraisal of cubist paintings. Br J Psychol 81: 483–495.

[90] Berlyne D (1970) Novelty, complexity and hedonic value. Percept Psychophys 8: 279–285.

[91] Henderson JM, Chanceaux M, Smith TJ (2009) The influence of clutter on real-world scene search: evidence from search efficiency and eye movements. J Vis, 9(1).32: 1–8. 10.1167/9.1.32 19271902

[92] Chai XJ, Jacobs LF (2009) Sex differences in directional cue use in a virtual landscape. Behav Neurosci 123: 276–283. 19331451 10.1037/a0014722

[93] Kent AJ (2013) From a dry statement of facts to a thing of beauty: understanding aesthetics in the mapping and counter-mapping of place. Cartographic Perspectives 73: 39–60.

